# General Anesthesia with the Use of SUPREME Laryngeal Mask Airway for Emergency Cesarean delivery: A Retrospective Analysis of 1039 Parturients

**DOI:** 10.1038/s41598-018-31581-5

**Published:** 2018-08-30

**Authors:** Xiaobin Fang, Quansheng Xiao, Qianling Xie, Ren Liao, Tao Zhu, Shiyang Li, Zhenyan Bo

**Affiliations:** 1 Department of Anesthesiology, West China Hospital of Sichuan University, Chengdu, 610041 China; 2 Department of Anesthesiology, Quanzhou women’s and Children’s Hospital, Quanzhou, 362000 China; 3 Department of Anesthesiology and Perioperative Medicine, Quanzhou Macare Women’s Hospital, Quanzhou, 362000 China; 4 Department of Anesthesiology, West China Hospital of Sichuan University, Chengdu, 610041 China; 5 Department of Anesthesiology, West China Hospital of Sichuan University, Chengdu, 610041 China; 6 Department of Anesthesiology and Perioperative Medicine, Quanzhou Macare Women’s Hospital, Quanzhou, 362000 China; 7 Department of Epidemiology and Biostatistics, West China School of Public Health, Sichuan University, Chengdu, 610041 China

## Abstract

In comparison to elective cesarean delivery, emergency cesarean delivery under endotracheal intubation is associated with higher risk of life-threatening airway problems. In this retrospective study, we evaluate the efficacy and feasibility of using SUPREME laryngeal mask airway (SLMA) in emergency cesarean delivery under general anesthesia (GA). The study included a total of 1039 paturients undergoing emergency cesarean delivery under GA with SLMA from January 2015 to December 2015 at Quanzhou Children’s and Women’s Hospital. Outcome measures included incidence of the adverse events related to using SLMA, maternal mortality, and neonatal outcomes. Briefly, no aspiration or regurgitation was noticed; the first attempt was successful in all but 2 subjects, both because of incorrect location, one was detected by decreasing oxygenation and the other by high airway pressure, the second attempt was successful in both cases. No subject was switched to endotracheal intubation. No laryngospasm or bronchospasm was detected. No maternal death occurred. There were 1139 neonates (including 944 single birth, 92 twins, 3 triplets) in this study, 5-min Apgar score was 7–10 in 1092 (96.72%) neonates. Thirty-seven (3.28%) neonates received endotracheal intubation. In conclusion, this retrospective study showed that the SLMA was used successfully in 1039 patients undergoing emergent cesarean delivery without any major complications. Vigilant attention by attending anesthesiologists is warranted.

## Introduction

Mainstream choice of anesthesia for cesarean delivery is neuraxial anesthesia. However, not all patients are suitable for neuraxial anesthesia for the reasons including patient refusal, coagulopathy, anticoagulant use, massive hemorrhage, placental abruption, uterine rupture, placenta accrete/percreta, cord prolapse with nonreassuring heart rate tracing, failed regional block, and so on. Moreover, emergency cesarean delivery often does not allow sufficient time for performance of spinal or epidural anesthesia. In these cases, general anesthesia (GA) is an acceptable choice^1^. The rapid sequence induction (RSI) is the preferred alternative when using GA for cesarean delivery^2^. However, difficult laryngoscopy is the most critical concern for using GA for cesarean delivery, which is associated with RSI^3^, especially in pregnant women who at risk of rapidly developing hypoxemias.

Laryngeal mask airway (LMA) has been used as a remedial airway after failed obstetric intubation^4–6^. LMA provides a reliable and rapid ventilation for parturients undergoing GA. The most critical issue in the use of LMA in cesarean delivery is the protection of the airway from regurgitation and aspiration^7,8^. The PROSEAL LMA (PLMA) and SUPREME LMA (SLMA) are designed to possess a double lumen to harbor a gastric tube through the mask. PLMA or SLMA have been safely used in the patients with high risk of aspiration like appendicectomy^9^, patients in the prone position^10^ and laparoscopic surgery^11^, and could provide protection against regurgitation and aspiration^12^. There are several recent reports of safety and efficiency with use of PLMA or SLMA for elective cesarean delivery^2,13^. In emergency cesarean delivery, patients often do not have enough fasting time. In our previous study^14,15^, no regurgitation or aspiration was found in a small number of patients receiving GA under SLMA for emergency cesarean delivery.

In this retrospective study, we present a total of 1039 mothers who underwent emergency cesarean delivery with the use of SLMA (Teleflex Medical. Approval Number: MMA36p) as airway management for GA, and report maternal and neonatal outcomes.

## Methods

### Ethics

The study protocol had been approved by the Quanzhou Women’s and Children’ Hospital Ethics Committee (Permit date: 20171126). Requirement for informed consent was waived. This study was conducted in accordance with the principles outlined in the Declaration of Helsinki, and the privacy of all subjects was protected. Only investigators had the permission to review personal medical records, and data anonymity was applied during data management.

### Study design and subjects

This study was a retrospective review including 1039 maternal who were used SLMA as an airway management for cesarean delivery under general anesthesia from January 2015 to December 2015 at Quanzhou Children’s and Women’s Hospital. In our hospital, the cesarean delivery rate is about 31%, under GA in a majority of the patients (about 90%), and the SLMA is used routinely^14,15^. During the study period, there were a total of 1486 cesarean sections, among which 1039 cases were emergency cesarean section, all using SLMA. The remaining 447 were elective surgery: 301 under general anesthesia using SLMA, and the remaining 146 with neuraxial anesthesia. The high rate of general anaesthesia for caesarean section in our institution was mainly due to patient preference.

Data of these 1039 women regarding relevant clinical information were retrieved. Demographic data included patient age, height, weight, body mass index (BMI), American Society of Anesthesiologists (ASA) physical status, cesarean scar pregnancies, multiple births, gestational diabetes mellitus (GDM), gestational hypertension (PIH) and other preoperative complications. Nurses involved in this study received specialized training for the use of SLMA. Data was retrieved from electronic medical records.

The main objective of this study was to identify the safety and effectiveness of the use of SLMA in cesarean delivery under general anesthesia. Outcomes related to safety and effectiveness were listed as follows:

1. Airway outcomes. These outcomes included: A. The rate of successful insertion of SLMA on first attempt. B. Incidence of changing or reinserting SLMA. C. The incidence of changing to endotracheal intubation.
2. Adverse events associated with SLMA. These event included: A. Rate of regurgitation and aspiration. B. Incidence of desaturation, laryngospasm or bronchospasm. C. Incidence of sore throat or dysphagia or hoarse voice.
3. Maternal mortality.
4. Neonatal outcomes. These outcomes included: A. 5-min Apgar score. B. Incidence of neonatal intubation, neonate mortality and morbidity.

Besides, operation and anesthesia related information was recorded.

The current study was retrospective in nature. As a result, there was no clearly defined protocol for the diagnosis. In our daily practice, successful insertion of SLMA was defined by the establishment of valid ventilation according to pulmonary auscultation and the presence of end-tidal carbon dioxide on capnogram. Regurgitation was defined as discovery of gastric content in the mouth, aspiration was defined by postoperative chest X-ray or chest CT upon suspicion, desaturation defined as pulse oximetry less than 93% for 1 min or longer, laryngospasm defined as complete obstruction of the airway with associated rigidity of the abdominal and chest walls, bronchospasm defined as increased respiratory effort, sore throat defined as continuous pain or discomfort in the throat independent of swallowing, dysphonia defined as difficulty speaking or pain on speaking, dysphagia defined as difficulty or pain provoked by swallowing. Maternal mortality was defined as the hospital-stay death of parturients after emergency cesarean delivery. Operating time defined as the time from skin incision to skin suture ends, anesthetic time defined as the starting anesthesia by intravenous to the time of patients leaving the operating room. Patients were routinely followed-up and asked about sore throat, dysphagia, or hoarseness in the postop period (3 days after the surgery) using a postoperative visit list that includes these items. Apgar score was recorded by a nursing staff.

### Anesthetic management

On arrival in the operating room, an intravenous access line was established to monitor heart rate and blood pressure. Patients also received continuous monitoring with electrocardiogram and pulse oximetry. After pre-oxygenation with 100% oxygen for 3 minutes, anesthesia was induced with intravenous propofol (2–3 mg/kg), cisatracurium (0.3–0.6 mg/kg) and fentanyl (50–100 μg). After disappearance of the eyelash reflex, SLMA (3# or 4#) was inserted. The cuff was inflated with 20–30 ml of air. Placement was verified with auscultation and the presence of end-tidal carbon dioxide on capnogram. Ventilation was carried out using a mixture of 50% N_2_O in oxygen and 2% sevoflurane. Inspired tidal volume was set at 6–8 ml/kg. Respiratory rate was 10–14 per minute. A 14 FG nasogastric tube connected with continuous suction was inserted via the gastric drainage aperture of the SLMA into the stomach, and gastric content was continuously suctioned during the operation. After the fetal delivery, myometrium injection of oxytocin (10–20 IU), a standard practice in our hospital, was performed routinely. Hemabate myometrium injection was based on obstetrician discretion. Sevoflurane was reduced to 1%, and maintained until the end of operation. SLMA and nasogastric tube was removed after the patient regained consciousness, could maintain adequate ventilation (tidal volume of 6–8 ml/kg, and respiratory rate of ≥12 per minute), and opened her eyes and mouth on command. All patients received routine opioid analgesia.

### Statistical analysis

Continuous variables are presented as mean (SD) and standard error (SE), and analyzed using Student’s t-test. Categorical variables are presented as count and proportion, and analyzed using Chi square or Fisher’s exact test. All data were analyzed using IBM SPSS 20.0 software (Chicago, IL, USA). P < 0.05 was considered statistically significant.

## Results

Demographic, obstetric and operative characteristics of the parturients are presented in Table 1.

**Table 1:** Demographic and obstetric characteristics of the patients.

Characteristics (n = 1039)	
Age (y)	29.11 ± 0.14
Height (cm)	158.13 ± 0.15
Body weight (kg)	67.48 ± 0.30
BMI (kg/m^2^)	26.98 ± 0.11
≥30 kg/m^2^	181 (17.4)
ASA II/III/IV	1005/33/1 (96.7/3.2/0.1)
Repeat cesarean deliveries (no, %)	510 (49.1)
Multiple births (no, %)	95 (9.14)
Gestational diabetes mellitus (no, %)	132 (12.7)
Pregnancy-induced hypertension (no, %)	74 (7.1)
Gestational hypertension (no, %)	26 (2.5)
Mild preeclampsia (no, %)	9 (0.9)
Severe preeclampsia (no, %)	37 (3.6)
Eclampsia (no, %)	2 (0.2)
Placenta previa (no, %)	71 (6.8)
Placental abruption (no, %)	39 (3.8)
Placenta implantation (no, %)	7 (0.7)
Duration of surgery (min)	34.46 ± 0.46
Duration of anesthesia (min)	45.41 ± 0.45
Time from skin incision to delivery (min)	5.47 ± 0.10
Time from induction to delivery (min)	10.56 ± 0.12

Values are expressed as n (%).

Data are presented as mean ± standard error or n (%).

The efficiency of airway and adverse events are presented in Table 2. The first attempt failed to place the SLMA correctly in 2 (0.20%) parturients, both due to incorrect location, one was detected by decreasing oxygenation and the other by high airway pressure. In both cases, second attempt was successful. No aspiration or regurgitation was recorded. No laryngospasm, bronchospasm, sore throat or dysphagia, or hoarse voice was detected. No maternal death occurred. None of the subjects were switched to endotracheal intubation.

**Table 2:** Efficiency of airway and adverse events.

Events (n = 1039)	
Successful placement on first attempt	1037 (99.8)
Change to intubation	0
Desaturation^#^	1(0.1)
Regurgitation	0
Aspiration	0
Laryngospasm or bronchospasm	0

Values are expressed as n (%).

^#^Desaturation is defined as pulse oximetry less than 93% for at least 1 min.

Basic information and outcomes of the neonates are summarized in Table 3. Neonatal endotracheal intubation was performed in 37 preterm neonates at 5-min after delivery and none of the term neonates. Among the 37 cases with endotracheal intubation, the gestation period was 34–37 weeks in 11 (29.7%)neonates, 32–34 weeks in 7 (18.9%) neonates (18.9%), and < 32 week in 19 (51.4%) neonates. One term neonate died within 24 hours after birth due to multiple malformations. Eighteen preterm neonates died within 24 hours after birth, of them 8 were stillbirths. No neonates died between 1 and 28 days. Apgar score at birth, 1-min and 5-min was 9.15 [95% confidence interval (CI), 9.07–9.23], 9.61 (95%CI, 9.59–9.66), and 9.82 (95%CI, 9.8–9.85), respectively. In term neonates (795/70%), Apgar score at born, 1-min and 5-min was 9.55 (95%CI, 9.50–9.61), 9.84 (95%CI, 9.81–9.87), and 9.95 (95%CI, 9.93–9.97), respectively. In preterm neonates (342/30%), Apgar score at born, 1-min and 5-min was 7.67 (95%CI, 7.48–7.86), 8.58 (95%CI, 8.45–9.72), and 9.18 (95%CI, 9.10–9.26), respectively.

**Tablee 3:** All Neonatal outcome.

All Neonatal outcome	All (n = 1137)	Premature birth^@^ (n = 342)	Term birth^@^ (n = 795)
Preoperative stillbirth	8(0.7)	8(2.3)	0(0)
Living neonates	**1129(99.3)**	**334 (97.7)**	**795(100)**
**APGAR at 5 min** ^*^			
0–3 scores	37^#^(3.28)	37^#^ (11.08)	0(0)
4–6 scores	0(0)	0(0)	0(0)
7–10 scores	1092(96.72)	297(88.92)	795(100)
**Postoperative outcome** ^**$**^			
Natal intubated	37(3.25)	37(10.82)	0(0)
Died within 24 h	11(0.97)	10(2.92)	1(0.13)
Healthy	1091(95.95)	297(86.84)	794(99.87)

Values are expressed as n (%).

^@^Term neonate is defined as born at or after 37 weeks of gestation, and preterm neonate is defined as born between 26 to 36 weeks of gestation.

^#^Natal endotracheal intubation was performed.

*N (%) mean the percentage in living neonates.

^**$**^N (%) mean the percentage in all neonates.

## Discussion

In this study the SLMA was used successfully in 1039 patients who underwent emergent cesarean delivery. The first attempt to insert the SLMA failed in only 2 cases, both due to incorrect location of SLMA, one was detected because of decreasing oxygenation and the other of high airway pressure. The second attempt was successful in both cases. No aspiration or regurgitation occurred. No laryngospasm or bronchospasm was detected. No maternal death happened.

Typically, intrathecal anesthesia has a low failure rate which is why it is often considered the anesthetic of choice for elective cesarean delivery by most international obstetric anesthesia organizations. For emergency cesarean delivery, however, intrathecal anesthesia may not be appropriate since longer time is needed to achieve satisfying anesthesia. SLMA is a modified single-use version of the PLMA. It preformed curved shaft consists of a double lumen with separate respiratory and alimentary tracts. The central lumen allows for access to the digestive tract using a gastric tube^13^. No aspiration or regurgitation occurred in the current study. When considered together with previous studies^2,13–15^, we believe that evidence is accumulating to support the safety of LMA for cesarean delivery. The successful rate of first insertion in this study is 99.8%. The high first attempt insertion success rate might be attributed to: the routine use of SLMA for general anesthesia in cesarean delivery at our hospital. All anesthesiologists in our department are experienced in using SLMA for parturients and familiar with the technical instruction recommended by manufacturers^14^. This successful rate is also comparable to that reported by more recent studies (97.7%-99.7%) when using LMA for airway management for cesarean delivery^2,13^.

For emergency cesarean delivery with general anesthesia, better outcomes depend on two components: rapid establishment of patent airway and avoidance of aspiration. The results of this study show both could be achieved with SLMA. We did not report the time required to the establish airway, but in previous studies^14,16,17^ as well as our experience, the time to establish airway with SLMA is 10–30 s. An extremely short time to establish efficient ventilation by SLMA was responsible for one important reason for satisfactory outcomes of parturients. Compared with RSI, which failed tracheal intubation was reported approximately 1:280 in the obstetric population^18^, SLMA seemed more beneficial because sufficient ventilation could be received in all patients in the study. On the other factor of concern for avoidance of aspiration, SLMA possesses a double lumen to allow a 14 FG gastric tube through the mask, the tube is connected with continuous suction, which can reduce gastric contents and intragastric pressure, decline greatly the potential risk of reflux and aspiration.

There were only 181 (17.4%) parturients with BMI ≥ 30 kg/m^2^ in the study. All underwent cesarean delivery safely with 100% successful rate of inserting SLMA at first attempt. Although proofs are not enough and further investigation is needed, this study may throw light on the use of SLMA for obese parturient.

Other adverse events associated with SLMA such as incidence of desaturation, laryngospasm or bronchospasm, sore throat or dysphagia or hoarse voice, was infrequent and slight according to previous literature^12^. We introduced those events as secondary outcomes although those symptoms may be subjective. Conducting SLMA meticulously and skillfully might lead to low risk of adverse events in this study, since general anesthesia with SLMA for cesarean delivery has been carried out for years in our institution.

Neonatal 5-min Apgar score and intubation was more associated with long-term outcomes^19^, we inducted them as second interest outcomes. As previous report^20^, a low dose fentanyl (50ug) exerts temporary depressant effects on fetal biophysical parameters at delivery. Albeit fentanyl was routinely used for induction and the time from skin incision to delivery was approximately 5 minutes, which may have the potential impact to neonates^21^, in a meta-analysis^22^, opioid interventions in parturient under GA affect neonatal at 5-min Apgar scores in neonates but appear no clinically meaning in safety, they could attenuate haemodynamic response for airway establishment and obstetric operation. It is a controversial practice but haemodynamic stability is required for parturient undergoing emergency cesarean delivery. In the study all term neonates had Apgar score ≥ 8 after 5 min, and none of them received intubation, one term neonate died within 24 h but was not considered to be the result of anesthesia. Endotracheal intubation was performed in 37 (3.28%) neonates, and all 37 intubation neonates were preterm, as the intubation rate of newborns in cesarean delivery was 0.09–6.79%^23^. The results of newborns in this study seem to indicate that GA with SLMA exerted no or transient influence on term neonates. However, for preterm neonate especial more vulnerable neonates, deliberate consideration and adequate preparation for neonatal resuscitation are prudent when GA for cesarean delivery is inevitable.

There are many limitations in our study. The study was performed in our hospital with the high rate of GA for cesarean delivery because of patient preference^14,15^. A retrospective study with a limited scale of 1039 participators and lacking a control group means finite persuasion. However, considered together with previous studies^2,13–15^ and results in the study, we believe that SLMA may be an alternative for emergency cesarean delivery when general anesthesia is inevitable. Our finding on use of SLMA in emergency cesarean delivery parturients seems more practical significance.

In conclusion, this retrospective study showed that the SLMA was used successfully in 1039 patients undergoing emergenet cesarean delivery without any major complications. Vigilant attention by attending anesthesiologists is warranted.
